# Visual Hallucinations in PD and Lewy Body Dementias: Old and New Hypotheses

**DOI:** 10.3233/BEN-129022

**Published:** 2013

**Authors:** M. Onofrj, J. P. Taylor, D. Monaco, R. Franciotti, F. Anzellotti, L. Bonanni, V. Onofrj, A. Thomas

**Affiliations:** ^1^Department of Neuroscience and Imaging, University G. d’Annunzio of Chieti-Pescara, ChietiItaly; ^2^Aging Research Center, “G. d’Annunzio” University Foundation, ChietiItaly; ^3^Institute for Ageing and Health, Wolfson Research Centre, Campus for Ageing and Vitality, Newcastle University, Newcastle Upon TyneUK; ^4^Università del Sacre Cuore, Istituto di Radiologia, RomeItaly

**Keywords:** Visual Hallucinations, Parkinson’s Disease, Lewy body dementias, default mode network

## Abstract

Visual Hallucinations (VH) are a common non-motor symptom of Parkinson’s Disease (PD) and the Lewy body dementias (LBD) of Parkinson's disease with dementia (PDD) and Dementia with Lewy Bodies (DLB). The origin of VH in PD and LBD is debated: earlier studies considered a number of different possible mechanisms underlying VH including visual disorders, Rapid Eye Movement (REM) Sleep Intrusions, dysfunctions of top down or bottom up visual pathways, and neurotransmitter imbalance.

More recently newer hypotheses introduce, among the possible mechanisms of VH, the role of attention networks (ventral and dorsal) and of the Default Mode Network (DMN) a network that is inhibited during attentional tasks and becomes active during rest and self referential imagery.

Persistent DMN activity during active tasks with dysfunctional imbalance of dorsal and ventral attentional networks represents a new hypothesis on the mechanism of VH.

We review the different methods used to classify VH and discuss reports supporting or challenging the different hypothetical mechanisms of VH.

## 1. Introduction

Historically, Visual Hallucinations (VH) in Parkinson's Disease (PD) first received attention when several reports showed that chronic dopaminomimetic treatments could trigger their onset [1–5].

VH were thus initially considered to be a drug-induced phenomenon, and were classified as "dopaminomimetic psychosis" or "levodopa psychosis" [3,4, 6–11], despite earlier reports showing that VH may occur as a consequence of anticholinergic treatments [12].

However subsequent studies demonstrated that the dose and duration of dopaminomimetic therapy are not major risk factors for VH nor do these phenomena relate to L-Dopa plasma levels [13–16]. Furthermore, a careful review of the literature of the pre-levodopa era, avoiding the confounding factors of dopaminergic stimulation and drug treatments, has confirmed that VH are an inherent feature of PD [17–26].

Thus rather than a simple sequela of dopamine dysfunction alone, VH appear intrinsic to PD in itself and have significant neuropathologic basis: there is now a significant corpus of data [27–30] which indicates that the strongest predictor of VH is the presence of neocortical alpha-synuclein aggregates (Lewy Bodies or Neurites) in the brains of patients with parkinsonism [31, 32]. Indeed as a consequence it has been proposed that VH as a non-motor symptom of Parkinson's should be

ISSN 0953-4180/13/$27.50 © 2013 – IOS Press and the authors. All rights reserved added as a supportive criteria to the operational clinical criteria for the diagnosis of PD [31].

From an epidemiological stand-point, VH are a common symptom in PD and increase with the duration of the illness; for example in the longitudinal study of Goetz et al. [33] of non-hallucinating PD patients at baseline at 4 years 26% had VH, 47% at 6 years, 47% and 60% at 10 years. VH prevalence in the Lewy body dementias (LBD) which include dementia with Lewy bodies (DLB) and Parkinson's disease with dementia (PDD) are even higher (up to 80%).

This association of VH with cognitive impairment is also true in non-demented PD patients with VH as these patients cross-sectionally tend to have greater impairments than non-hallucinators across multiple cognitive domains, including, for example, visuospatial/visuoperceptual function as well as frontal functions such as sustained attention, response inhibition and verbal fluency [34–39]. The occurrence of VH are also an important predictor of future cognitive decline and consequent dementia in PD [22,40–42] and are frequently co-associated with the occurrence of other neuropsychiatric symptoms [30,43,44].

Thus, in addition to having deleterious effects in themselves, VH with their co-association with cognitive dysfunction, dementia and other neuropsychiatric symptoms have a significant on the quality of life of patients with PD or LBD and their care-givers [45].

Clearly there is a clinical need to understand the aetiology of VH in PD and the LBD. However despite neuropathological associations between alpha-synuclein deposition and VH occurrence [27–32] in limbic and fronto-temporal-parietal cortex, their underlying pathophysiology is still poorly understood.

Nevertheless with the present paper we aim, using new evidences from our own group and others to discuss some possible mechanisms of VH as well as suggest future areas for research.

## 2. Classifications

Before considering VH in PD and LBD we propose a reminder of the positive spontaneous visual phenomena, which are also classifiable as simple or minor VH (Table 1). In addition, it is also important to reflect upon two classic syndromes presenting with VH, Charles-Bonnet syndrome (Box 1) and Peduncular hallucinosis (Box 2) as the phenomenology and proposed aetiologies of these have implications for understanding visual hallucinations in PD and LBD.

**Box 1** *An archetypal clinical model of visual hallucinations: Charles-Bonnet Syndrome.*
Charles Bonnet Syndrome (CBS) patients display VH associated with significant visual loss, with the latter arising as a consequence of cataracts, maculopathy, and optic neuropathy [149–151]. Specific tassellopsies, teicopsies and dendropsies are described in 90% of patients with CBS [152]. More complex hallucinations are also frequent: hallucinations of faces affect 41–47% and hallucinations of people-like figures affect between 40–71% of patients. Hallucinations in this syndrome are traditionally said to be Lilliputian in character [45], although it is now evident that, like in PD, such miniaturization appears only in a minority of CBS patients [54]. A consistently reported feature is preserved insight into the unreality of the hallucinations, but patient interactions with the hallucinations can occur and 41–59% of patient report a strong emotional salience with the occurrence of these hallucinatory phenomena.
VH in CBS has been simplistically considered to be arising as a result of bottom up deafferentation. However the aetiology of VH in CBS as being simply due to visual loss is currently debated [153] and indeed loss of vision cannot be the sole explanation, as the majority of patients with visual impairment do not experience hallucinations, and hence, other factors are likely to be contributory [153].

**Box 2** *An archetypal clinical model of visual hallucinations: Peduncular hallucinosis.*
Peduncular Hallucinosis (PH) appears with lesions involving one of the cerebral peduncles and at least two reports describe hallucinations and hemiparkinsonism contralateral to the lesion [154, 155]. VH are typically complex and vivid, and "although insight is preserved and patients recognize the unreality of the images" [156], the vivid character of the VH is said at times to have a profound emotional effect on the patient [155,157]. In contrast to CBS, tassellopsies and dendropsies are not seen in PH. In PH the content of VH consists of inanimate objects, but mostly complex kinematic scenes are described. For example, in a report by one patient the room was transformed into a train carriage and then into a train with people walking and airplanes flying from the ceiling [71]. As a theoretical model for VH it is hypothesized that VH in this disease arise from brainstem dysfunction and depend upon Rapid Eye Movement Sleep (REM-S) regulating structures.

## 3. VH in PD and Lewy body dementias

VH are by far the most common hallucinations in PD and LBD although acoustic [46] and haptic (tactile) hallucinations are reported in variable percentages of patients (11–19%). Olfactory hallucinations have also been described recently and open to debate is the question whether somatic complaints which are often reported by patient with PD and DLB [47] actually represent a form of somatic hallucinations.

In terms of phenomenology, VH in PD are usually "formed" from the very first appearance of the hallucinatory symptoms. Various descriptions report inanimate objects (leaves on the wall) [48], animated figures (children playing) [48], normal size figures [16,17, 49,50], miniature people and animals [45,51], images from daily life experience [16,52], and images from TV programs [16].

Tassellopsies, dendropsies, fortifications spectra and visual distortions such as micro-macropsies, telopelopsies are never described in PD although polyopic phenomena might explain some of the diplopias intermittently reported by PD patients.

Typical of PD are early blurred hallucinations consisting of sensation of presence, which have also been called "extracampine hallucinations" [53] because the presence is felt at the extreme peripheral border of the visual field, or at one's shoulders.

Blurred hallucinations can also consist of sensation of sideway passage (mostly animals or shadows fleeing at the subject sides) [16].

VH tend to become more complex and severe in PD when cognitive impairment manifests [16], and indeed in DLB often the fully formed complex VH are the first visual symptom to be reported rather than the presence or passage hallucinations which are more typical of PD.

In patients with cognitive decline as well as the incidence/prevalence of hallucinations being higher, the hallucinations are also qualitatively different, and complex interactions with hallucinatory perceptions are described due to the lack of insight into the unreal nature of perceptions [16,17,43,54–57]. Descriptions in the literature have included the patient who interacts with "devils with blurred faces and changing size armed with blades" [16] or quarrels with the hallucinatory presence molesting his wife [58].

In patients with PDD or DLB, night-time hallucinations can assume the form of confusional states, defined as oniroid (oniric) confusion, status dissociatus or agrypnia excitata [59]. In this confusional state, the patient interacts with VH and wandering behaviour and agitation are common.

In contrast night-time confusional state in PD is a poorly described condition. It is not clear how frequent it is, as reports are only anecdotal [48], nor is it clear if the nocturnal confusion with VH is related to REM sleep Behaviour Disorder (RBD). In brief, RBD is a parasomnia consisting of the loss of normal muscle inhibition during sleep and enacting of dreams. During enactments the patients might fall off the bed or hurt their partner with their uncontrolled movements or by kicking and punching induced by the content of their dreams [60]. RBD is frequent in PD patients, and its occurrence may precede the appearance of motor symptoms by many years [61]. RBD occurrence statistically predicts the occurrence of dementia of the synucleinopathy type [62]; in DLB it is considered one of the strongly supportive symptoms for the diagnosis of condition [63], and is considered as one of the possible causative or precipitating factors for the occurrence of VH [62,64]. However accurate identification of RBD on polysomgraphy can be difficult in PDD and DLB patients as frequently these patients have concurrent abnormal slow wave EEG activity [63] which makes it difficult to see the REM desynchronisation. This, with the observation that slow wave activity in itself is associated [63] with confusion and cognitive fluctuations makes it hard to determine whether nocturnal confusion in DLB/PDD is actually an extreme example of RBD.

## 4. Assessment of visual hallucinations

Understanding the underlying neurobiology of VH in the Lewy body diseases is dependent upon having a clear phenomenological description of these subjective phenomena and concerns have been expressed, particularly in PDD and DLB patients that due to cognitive dysfunction which includes executive impairment and lack of insight that patients may not be adequately able to describe these internally generated phenomena. However a recent phenomenological analysis, performed in PDD and DLB patients, showed that cognitively impaired patients were able to report the experience of mostly daily complex hallucinations, and their duration which typically lasted minutes. Most patients commonly saw people or animals and the experiences were usually perceived as unpleasant [57]. Neuropsychiatric symptoms coexisting with hallucinations were apathy, sleep disturbances and anxiety [57]. The conclusion of these authors was that patients with mild-to-moderate dementia can provide detailed information about their hallucinations and thus studies probing aetiological mechanisms of VH in PDD and DLB can be reassured that the subjective reports of patients of their experiences can be reasonably relied upon, certainly in milder cases.

With Table 2 we propose a method to categorize hallucinations in PD and related disorders in order to allow quantification studies. There are a number of rating instruments for VH: the most used is item B of the Neuropsychiatric Inventory Worksheet [65]. In this scale, however, acoustic, optic and olfactory hallucinations are rated together in a scale of frequency and severity (distressing or not). Thus while it is an useful instrument, yet it is not specific to PD or DLB hallucinations, which are complex, progressive, and not to-

Table 2
Categorization of visual hallucinations

| Parameter | Measurement | Rationale |
|---|---|---|
| Cognition | Dimensional score or catergorisation typically as either mild, moderate or severe impairment | Reported so far. Quantitative assessments with scales (e.g. MMSE, ACE-R, CAM-COG, and DRS 2) should accompany categorization of patients. |
| Influence of focused attention | Effect leads to disappearance or not of VH | A frequent feature of VH in PD patients is the disappearance of the object of the hallucination under focused attention. This quality of VH has implications on the possible origin i.e. VH mediated via attentional dysfunction. |
| Insight | Preserved<br>Lost | Preserved or reduced insight on the non real nature of VH is prognostic factor, as shown by classifications in benign/malignant forms. Abnormal insight predict the development of further behavioral disturbances. |
| Emotional content | Absent<br>Present | Fear, interest, curiosity, associated feelings (e.g. bothersome, haptic hallucinations) or the absence of emotional interaction with detachment should be reported. |
| Quality | Formed, not formed<br>Blurred, detailed<br>Static, kinetic/kinematic | May suggest mechanistically different aetiological sources of the VH although as yet we lack an understanding of these processes. |
| Frequency | Occasional, recurrent (daily, weekly, monthly) | Useful metric for assessing treatment response. |
| Duration | Seconds<br>Minutes<br>Hours | May relate to attentional dysfunction and to cognitive and motor fluctuations. |
| Time of the day when VH occur | Serotine<br>Daytime<br>Night time | In cognitively preserved PD patients, VH are usually serotine. |
| Relationship with drug treatment | Effect of withdrawal or reduction in dose<br>Effect of introduction or increment in dose<br>Effects of pharmacological treatments | Besides historical approach to “dopaminergic psychosis” only few reports state that VH are increased by dopamine agonists. The relationship with non dopaminergic treatments including cholinesterase inhibitors, needs to be investigated. Clarification of anti-cholinergic effects of any drugs prescribed is also relevant as these may affect cognition and VH occurrence. Other specific agents which can induce VH include tramadol and memantine (although the latter may anecdotally improve VH). Antidepressant agents may also induce hallucinations (rarely) or RBD (frequently). |
| Presence of Visual disorders | Standardized scales | |
| Presence of Sleep disorders | Report all drugs used | The role of sleep disorders is described in the text. |

tally understood. For this reason we propose a quantitative scale, mostly focused on VH and indeed alternative scales which have greater utility in this regard include the Mayo visual hallucination inventory [66], the Queen’s Square visual hallucination inventory [67] and the Newcastle Visual Hallucinations Inventory [68]; the latter explores the phenomenology, frequency and emotional impact of visual hallucinations and also the co-association of hallucinations in any other modality with the opportunity for subjective description of any visual phenomena. There are also patient and carer variants of the questionnaire which enhance the reliability and validity of hallucinatory reports.

## 5. Mechanism of hallucinations

### 5.1. Disinhibition phenomena

A release phenomenon (i.e. disinhibition of neural structures, with increase in the excitability and spontaneous activity of the disinhibited neurons) is one of the core hypotheses in the aetiology of hallucinations. This mechanism is used to explain VH in CBS [30,31,69] and classic migraine. With release, it is proposed that tassellopsies and dendropsies, fortification spectra or heat waves are caused by spreading depression inhibiting the idiotypic cortex and “releasing” unimodal association areas. Tassellopsies and dendropsies in CBS and fortification spectra in migraine have specific orientation and spatial frequency characteristics that suggest a disinhibition of columnar structures in primary or associative visual cortex [70]. Based on the disinhibition theory, VH are thought to be caused by phasic increases in neural activity within the specialized visual cortex, and the type of VH is defined by the specific location of the increased activity. Table 3, based on the seminal papers by ffytche et al. [71–73] summarizes anatomical areas involved in the generation of different types of VH: coloured hallucinations are accompanied increased activity in V4 [74]; hallucinations of land-

Table 3
Cortical Areas involved in production of Visual Hallucinations

| | |
|---|---|
| V1 V2 V3 | Dendropsies<br>Tassellopsies<br>Teicopsies<br>Heat Waves<br>Macro or micropsies |
| V4 | Colors |
| V5 | Moving (blurred) VH |
| Anterior ventral temporal lobe | Landscapes<br>Vehicles<br>Objects |
| Superior temporal sulcus | Faces<br>Face metamorphopsies |
| Cuneus and precuneus | Palinopsies (visual perseverations) |

scape figures, vehicles and various other objects are accompanied increased activity in the anterior ventral temporal lobe (anterior to fusiform and lingual gyri; projections of the ventral visual pathway); hallucinations of faces (or metamorphopsia of faces) associate with hyperactivity in superior temporal sulcus; and increased activity in the parietal cuneus and precuneus areas (receiving projections from the dorsal visual pathway, where the reference frame that ensures the stability of the visual world across successive eye movements is located) is accompanied by visual perseveration, autoscopic phenomena and palinopsia (because of shifts of the reference frame) [73,75].

The disinhibition mechanism also implicates cholinergic denervation in the origin of VH. This hypocholinergic hypothesis, with the ensuing release/disinhibition phenomenon, has been supported in an extensive review [76] and this hypothesis suggests that cholinergic denervation of visual associative areas induce hallucinations because of a release phenomenon: cholinergic projections from the basal forebrain reach visual associative areas with their axon terminals [76]. In DLB and in PDD patients, it is well established that there is a reduction of cholineacetylase and cholinesterases in cholinergic nuclei of the basal forebrain and in cortical areas receiving afferent neurons from these nuclei and that these deficits are more prominent than those seen in AD [12,77,78]. A therapeutic trial with the cholinesterase inhibitor rivastigmine showed that hallucinations could be reduced in PDD patients by the administration of this drug [79,80]. Conversely anticholinergic drugs have a predilection for causing VH [81], particularly in individuals with pre-existing cholinergic deficit.

### 5.2. *Visual disturbances*

Pre-geniculate visual disturbances and eye problems (e.g. dry eyes, contrast sensitivity, diplopia, loss of acuity, loss of motion and colour perception etc.) are part of non-motor symptoms in PD [82] and many earlier studies have reported abnormalities of pattern electroretinograms (ERGs) and visual evoked potentials (VEPs) and abnormalities of visual perception in PD patients [83–88].

Dopaminergic dysfunction has been implicated as a cause: retinal dopaminergic cells modulate contrast sensitivity of retinal ganglion cells and hypodopaminergic states can be accompanied by blurring of spatial contrast, which are reversed by dopaminergic drug administration [86].

Empirical evidence for these comes from a variety of experiments: 1) Animals treated with MPTP or dopamine blockers/depleting agents confirm the dopaminergic effects on the visual system [89–91]; 2) Post-mortem neuropathological evidence demonstrating a primary deficiency of retinal dopaminergic circuitry in PD with eosinophilic and ubiquitine stained inclusions in the retina [85,86,91–93] with abnormalities in PD attributed to dysfunctions of retinal dopaminergic cells (amacrine and interplexiform cells) [92].

Visual abnormalities lead to changes in ERG and VEPs. In patients with PD, ERGs have smaller amplitudes and VEPs have delayed latencies. These alterations depend specifically on the spatial frequency and contrast [84,90,94,95] of visual stimuli. The retinal dopaminergic dysfunction alters the perception of spatial frequency characteristics of perceived images and reduces meaningful information for central visual processing: finer details of visual stimuli are blurred and contrast and colour discrimination [96] are reduced.

Both ERG and VEP alterations can normalize after patients receive L-Dopa or apomorphine [84,85,89,90, 97]. Visual perception, as well as ERG and VEPs amplitudes and latencies can change over time in parallel with “on” and “off states, with patient’s visual function improving when they enter “on” states.

In one of our studies we investigated whether VH or illusions might emerge in hypodopaminergic conditions, such as at the end of sleep benefit and during off phases [90]. In a series of different virtual reality experimental sets, we found that illusions, consisting of emergence of animated or human figures or inanimate objects, were more frequent during hypodopaminergic states [90]. A more detailed description is described in Box 3.

**Box 3** *Generating visual hallucinations in Parkinson's disease patients in a virtual reality experimental setting.*

The results of an experimental study showed a time relationship between VEP abnormalities, visual hallucinations (VH) and worsening of motor performance. PD patients were placed in a virtual environment to restrict sensory input to visual input only. Exploration and pointing time, avoidance of obstacles were evaluated in virtual environments reproducing daily living situations (supermarket, gymnasium, kitchen). Patients underwent one 35 min session of virtual reality experience in each of the three selected environments presented by SONY Grasstron Plm-Ass-goggles with an Inter Trax 30 serial gyroscopic tracker.

Twenty-three patients with idiopathic PD according to UK Brain Bank Criteria (mean age 65.3 ± 7.7 SD, disease duration 5.2 years) and 15 patients with non parkinsonian neurological disorders (6 postictal hemiparesis, 9 polyneuritis), mean age 67.1 ± 4.9, without a history of VH, did the virtual exploration in the early afternoon, while receiving their standard dopamine replacement therapy. In this experimental condition none of the control patients experienced visual dysperceptive phenomena but six patients who experienced medication wearing-off fluctuations described visual elements which were not present in the virtual environment. These six fluctuating patients underwent a second experimental protocol: the immersion in the same virtual environments was repeated four times, separated by one week. At week 2 and 4 subjects were tested in the early morning after night time sleep, without taking the early morning treatment dose and at weeks 1 and 3 they were tested with the usual morning antiparkinsonian therapy. Visual evoked potentials (VEPs) were recorded in these six patients before and after the virtual environment immersion. All the six patients, in short-term drug deprivation conditions (weeks 2 and 4) described misinterpretations of the elements presented in the explored environment and described images definitively not included in the virtual reality. In contrast no visual dysperceptions were experienced when the morning treatment doses were taken normally (weeks 1 and 3).

These images, either phenomenologically visual illusions or hallucinations consisted of human figures, animals and objects (for example, people in the gym, a gas pump in the kitchen) and appeared 50–80 min after patient wake-up, and 5–15 min after the virtual reality experimental session began. They lasted 3–25 s, and were accompanied by motor deterioration (the Unified Parkinson's Disease Rating Scale (UPDRS) motor score was 12 ± 3 before the virtual environment recording session, and increased to 22 ± 3 at the end of the session), thus suggesting that they coincided with the end of the sleep benefit.

VEP latency (to 2 cycles per degree grating, 15' arc grating width) was 116.4 ms before the recording session, and increased to 129.6 ms at the end (an increase of 7–38 ms).

We concluded that these visual experiences or VH appeared when the benefit of drug treatment was fading, for example in "off" phases in the first experimental protocol or when the sleep benefit ended, in the second; the latter is attributed to restoration of dopamine vesicular content during night time [55], and its effect lasts no more than a few hours, in accordance with the decay time of dopamine molecule per vesicle and with the number of released vesicles per minute.

Our experiments using the virtual environment therefore show visual experiences can differ in patients off and on medications.

The timing of the type of VH elicited by the virtual environments coincides with the timing of motor deterioration and the occurrence of VEP alterations, reflecting visual abnormalities. Four of the 6 patients complained of simple or complex VH in the 14–15 months follow-up after the virtual environment study.

The onset of hallucinations during "off" states and at the time when sleep benefit ends therefore shows that in PD, a certain type of VH may be related to low dopaminergic activity.

This study suggests an original and unique approach for studying VH and producing them in a manner which allows experimental evaluation. In an artificial context, the recognition of visual images not generated by the system, could be rated as VH. Despite this, several criticisms of the method can be made including the possible induction of VH because of decontextualization and because of the artificial simplification of visual stimuli. Nevertheless the method may be a promising approach for the evaluation of VH and warrants further research. For example, in patients affected by PD or by different forms of dementia, the virtual reality method might be used to obtain fMRI recordings of activities during VH with the comparison of brain activity for externally and internally produced images allowing for a better disentanglement of involved brain areas.

Recently a similar method was used for the assessment of VH predisposition in DLB. In this study [158] different ambiguous images were presented and patients were asked to describe images: incongruent descriptions were interpreted as suggesting the occurrence of paraidolias and thus as evidencing predisposition to VH. A similar method was used to study oltactory hallucinations [159].

Therefore in patients receiving adequate doses of L-Dopa or dopamine agonists (DAs), ERGs and VEPs can return almost to normal whereas in patients "off" therapy they again become abnormal.

Yet, pre-geniculate visual disturbances cannot thoroughly explain the emergence of VH as hypodopaminergic visual disturbances (and the accompanying abnormalities of visual evoked potentials and electroretinograms) [98,99] are corrected by transient or chronic administration of dopaminergic therapy [64], whereas hallucinations mostly appear in chronically treated PD patient [1–7]. In addition, despite the occurrence of visual abnormalities in PD, ocular pathology doesn't seem to demarcate PD patients who hallucinate from those compared to those who did not [30]. Beyond this, a second reason to dismiss the simplistic hypothesis suggesting that VH in PD are dependent on pregeniculate visual dysfunction is the fact that hallucinations in PD do not only occur in the visual modality but are also apparent in other sensory modalities, albeit, less frequent suggesting that cortical pathophysiological changes have a greater role.

### 5.3. *Disturbances in visuo-cortical areas*

Numerous lines of evidence now support visuo-cortical dysfunction as a contributor to VH occurrence in PD and the Lewy body dementias. Lower visual areas (e.g. V1 through to V3) appear to be less implicated as: 1) there appears to be a perseveration of these areas both at a macro and micro-structural level [100–102], for example, in DLB; 2) perfusion and functional imaging have suggested visuo-perceptual deficits [103] and VH are associated with higher visual areas [104, 105] and the threshold of occipital transcranial magnetic stimulation (TMS) induced phosphenes, a marker of lower visual area cortical excitability, appears similar in DLB to aged controls [106]. The authors of the latter observation reported an association between visual cortical excitability with TMS and the frequency and severity of VH in DLB and they provocatively suggested that the intrinsic excitability level of lower visual areas may be inherent to the individual's 'premorbid' neurobiology and that differing phosphene thresholds may contribute to the predilection in some individuals towards VH when they develop alpha-synuclein neuropathology.

Beyond this a number of fMRI studies in PD have suggested that patients with VH show decreased activity in extra-striate and visual association areas as well as frontal involvement thus implicating both bottom-up dysfunction as well as top-down and attentional processes in the generation of VH [105,107,108]. Abnormalities within the ventral visual stream as evidenced by increased temporal lobe Lewy body pathology [27, 30] and white matter disruption to the inferior longitudinal fasciculus [109] in LBD patients with hallucinators compared to non-hallucinators also support the role of bottom up dysfunction; conversely increased atrophy [110] and Lewy body pathology within frontal lobes [30] of LBD patients with hallucinations compared to those without support a top-down role in VH. Overall these observations provide empirical support to a number of models of VH which are discussed below in more detail.

## 6. Dream overflow and sleep disorders

Several studies postulate links between VH and sleep disturbances. Historically Moskowitz et al. suggested that altered dreaming is the first step in a progressive cascade of events leading to drug induced psychosis, mediated through a mechanism involving pharmacological kindling [3]. Pharmacological kindling now appears controversial; nevertheless the association between sleep disturbance, such as altered dreaming and sleep fragmentation [55,111] and VH has been suggested with these sleep disturbances considered predictive or coincident factors needed for the occurrence of treatment-induced VH. Arnulf et al. [112] and Comella et al. [113] argued that treatment-induced VH represents the intrusion of REM sleep into wakefulness, and that VH represent a dream overflow phenomena. Certainly more recent evidence has specifically established a link between VH and RBD [28,30,113,114]. In our chronic long-term study, RBD was an independent predictor of VH occurrence [54] and RBD and VH were related to the amount of DA agonists administered: patients who experienced both RBD and VH were receiving bromocriptine equivalent 32 $\pm$ 6 mg/day, while those with neither RBD nor VH were receiving bromocriptine equivalent 11 $\pm$ 6 mg/day ($p < 0.001$).

Yet, our study described the occurrence of VH in a selected population in which patients with dementia (PDD or DLB) were specifically excluded. The prevalence of VH in our patient study group was less than three-quarters the prevalence reported in other studies (40–47%) where cognitive decline signs were not among exclusion criteria. As we included only PD patients who did not develop any cognitive impairment during follow-up, our choice might have represented a selection bias. Thus a strict relationship between RBD and VH has yet to be demonstrated and this is supported by the 10-year longitudinal study by Goetz et al. [115] who found that while there is a co-association between VH and sleep disorders (e.g. sleep fragmentation, vivid dreams/nightmares, acting out of dreams), the presence of sleep disorders did not predict VH development. Interestingly, a recent longitudinal study [62] confirmed our earlier results that VH and fluctuating cognition is associated with RBD; nevertheless the origin of this association needs further elucidation.

Indeed while RBD is now considered to be a prominent non motor symptom of synucleinopathies [116, 117], and its role in PD and DLB might justify the ascending neurodegeneration hypothesis [118,119], proper studies on the relationship between RBD and VH in PD, DLB, PDD have yet to be designed and conducted. As anticipated in the phenomenology section, the link between RBD and nocturnal confusional states is also far from being understood, and the main question would be whether VH, RBD and nocturnal oneiric confusion are distinguishable entities or part of a continuum of symptoms subserved by the same mechanisms.

Despite the unclear nature of associations, the interpretation of VH as dream over flow phenomena in PD is still considered by some to be a major aetiological mechanism by which to explain VH. One study, for example, analysed hypocreatin (Hcrt) levels in PD patients, because of the similarity between symptoms such as sleep attacks, nocturnal insomnia, RBD, VH and depression that are consistently reported in PD [54,55,59,111,114,120–127] and in narcolepsy [128], which is linked to a selective loss of Hcrt neurons. Despite suggestions that VH in PD are an expression of symptomatic narcolepsy [112], the similarities between narcolepsy and PD non-motor symptoms are called into question by the observation in one of our studies that the DRD2 haplotype, which is a hallmark of narcolepsy, is not associated with VH and RBD in PD patients [125].

In final comment, the frequent falls [117] of DLB patients have been attributed to cataplectic attacks – part of the narcoleptic syndrome – due to liberation or overflow from REM sleep control of the motor inhibition commonly accompanying REM sleep, thus adding cataplexy to autonomic dysfunction as one of the causes of frequent falls in these patients. While recent studies found a correlation between dysautonomia and VH [129,130] extended polysonnographic monitoring has failed to confirm that REM sleep intrudes in waking state of PD patients with VH [114], a finding at variance with what is observed in patients with narcolepsy-cataplexy. Furthermore, there are differences in the phenomenology of dreams and VH [131]; while PD patients during RBD are actually dreaming, they do not interact with observers and often do not remember their dreaming experience, yet the same patients during VH can remain interactive with vivid recall of these hallucinatory events.

Therefore in summation we would contend that RBD dream overflow in Lewy body diseases is less likely to be a significant contributor to the aetiology of VH in these conditions.

## 7. Further models

More recent studies proposed different integrative models for the origin of VH in PD. One model described by Diedrich et al. [49] encompassed the occurrence of VH inside Hobson's schemata of mental states [121] where states of cerebral activity are schematised in the so-called Activation-Input Source-Modulation (AIM) model. Activation reflects the rate at which the mind can process information, input source is a measure of the level to which the brain processes external sensory data (in waking) or internal perception or information and modulation is the ratio of aminergic (noradrenergic and serotoninergic) predominance in waking to cholinergic predominance in REM sleep. Based on that model, hypnagogic and hypnopompic hallucinations, associated with transitions into and out of sleep, respectively, result from the REM-like enhancement of internal stimuli together with an aminergically activated waking brain. In this integrative model multiple mechanistic processes can contribute to the appearance of VH including visual impairment, reduced activation of primary visual cortex, abnormal activation of associative visual and frontal cortices, lack of suppression or spontaneous appearance of internally generated images through the ponto-geniculo-occipital system, intrusion of dreams into wakefulness, impaired filtering capacities of the brainstem, and over-activation of mesolimbic systems [48].

Another similar interactive model of VH was proffered by Collerton et al. [132] called the "Perception and Attention Deficit (PAD) Model" was used to describe the origin of VH across many disorders. In essence this model proposed that a combination of poor attentional binding (top-down) and perceptual impairments (bottom-up) causes, in a context specific visual scene, the intrusion of an expected but incorrect perception (hallucination) whose unreality is then not challenged due poor perceptual function.

In relation to these models, a third model of VH, specific to PD and Lewy body dementias [64] has focused on the role of frontal lobe in the control of focalized attention. Clinically a frequent feature of VH in PD patients is the disappearance of the object of the hallucination under focal attention [59]. Patients with significantly preserved cognitive functions spontaneously report that the hallucinatory images disappear when they try to focus their vision on the object. Focusing attention on VH in order to make them disappear is described as a "coping strategy" or non-pharmacological management approach for dealing with VH [133].

Thus the effect of focal attention on suppressing VH of PD patients implies a central role of attention for understanding the aetiology of VH in PD.

Attention is modulated through dorsal and ventral frontoparietal networks. When focusing on or perceiving images it has been hypothesed that a pre-attentive process scans a feature map that preserves memories of previous images in parieto-temporal brain regions and which receive afferent input from the dorsal and ventral visual pathways [134]. The feature map is processed in a master map (attentional matrix) or saliency map that identifies, in a preliminary fashion, conspicuously noticeable elements from the pre-attentive scan. Following this, salient elements are analyzed in detail by spotlight (focal) attention with the pulvinar, claustrum, superior colliculus and prefrontal cortex acting as the anatomical substrates of the master map and the spot-

Table 4
Resting state and attention control networks

| | Function | Cortical areas |
|---|---|---|
| Default Mode Network (DMN) | Interpretation, self referential, "internal conversation", imagery | Posterior Cingulate Cortex Precuneus<br>Medial Prefrontal Cortex<br>Medial Temporal Lobe<br>Lateral and Inferior Parietal Lobe |
| Ventral Attention Network (VAN) | Attention to salient stimuli<br>Modulates activation of other networks | Right Basal-lateral Amigdala<br>Ventral Frontal Cortex<br>Right Temporoparietal Junction<br>Ventral Striatum |
| Dorsal Attention Network (DAN) | Voluntary Orienting<br>Cognitive Information processing | Caudate (head)<br>Dorsolateral Prefrontal Cortex<br>Superior parietal lobule |

light of attention [105,135]. Not unreasonably, therefore, the frontal lobe dysfunction observed in PD patients might lead to an abnormal organization of the saliency map and spotlight attention which concurs with evidence suggesting neuropathologic, functional and atrophic changes in the frontal cortices which are associated with VH (as per previous section). Thus in this model, by focusing on attention effect and attention pathways, the core mechanism for VH occurrence in PD is moved from subcortical (arousal and sleep regulating brain stem structures) to cortical dysfunction.

However if dysfunction in fronto-attentional processing was the pre-eminent contributor to VH, then one might expect that VH should arise in other conditions associated with frontal dysfunction, for example attention-deficit disorder, or in those individuals with frontal lobe lesions, which is not typically the case. One explanation is that it may that there is something particular about frontal Lewy body pathology that predisposes to VH in PD and LBD. Alternatively, and more likely, it may be that frontal dysfunction is not the sole determinant; rather alterations in distributed functional brain networks which encompass frontal and other cortical areas may be more relevant i.e. the interaction between top-down and bottom up processing which returns one back to integrative models proffered by Diedrich et al. [49] and Collerton et al. [132].

In this context, more recently widely distributed resting state networks have provided further evidences from which to formulate new cortical models of VH [136]. Perhaps most relevant are the default mode network (DMN) and attentional networks (dorsal attention network, DAN and ventral attention network, VAN). These networks modulate the rapid transitions of the brain from a resting to an active state, in response to salient stimuli which require executive processing. Specifically, activity in the DMN has been correlated with periods of "task-independent" internal thought (mind wandering). The network represents an ontologically developed resting state of the brain, during which the retrieval and manipulation of episodic or semantic knowledge occurs. The VAN is engaged in presence of salient stimuli, providing a bottom up information while the interacting DAN provides a top down information, processing the direction of attention and encoding neural signals related to the behavioral significance of stimuli. The associated anatomical substrates for these specific networks are described in more detail in Table 4.

From these networks Shine et al. [136] developed the hypothesis that VH in PD reflect the relative inability to recruit activation in the dorsal attention network in the presence of ambiguous percepts, leading to overreliance on default mode network processing and salience arising from the ventral attention network and thus reinforcing the generation of false images. This functional failure of the DAN was proposed to stem from the presence of Lewy Body ($\alpha$-synuclein) pathology and certainly several neuropathological [137] as well as structural and functional neuroimaging studies have confirmed atrophy [138], white matter abnormalities [139], alterations in perfusion/metabolism [140–142] and both Lewy body and amyloid depositions [143] in structures of the three networks of PD patients. Further support for the possible role of these networks in VH has come from a number of fMRI studies where the DMN was consistently found to be hyperactive or normally active in patients affected by DLB [144] or PD [145] at variance with its observed hypoactivation in AD. Specifically Sauer et al. [146] noted that DMN activity is increased in patients with DLB in comparison with AD patients [147] and Rektorova et al. [144] that DMN activity in PD is not reduced during engagement in a visual recognition task.

From our own group [148] we have recently demonstrated in AD, the overall activity of the DMN was lower than in controls and DLB, and that this reduction was mainly related to a decrease of activity in the Posterior Cingulate Cortex (PCC), while in DLB patients the engagement of the PCC was not altered. Additionally in DLB alterations were found in the inter-hemispheric left-right connection and in the connection between fronto-parietal areas in the right hemisphere. Secondary analyses performed by separating DLB patient with moderate and severe fluctuating cognition (fl Cog), showed that the PCC hub could be clearly identified even in severely fluctuating patients and thus the persistent activity of DMN was interpreted as the essential background to fluctuating cognition as well as linking arousal and attention fluctuations to the clinical phenotype observed in different forms of dementia. Despite the difficulties in assessing fluctuating cognition and attention and its relationship with abnormal perceptions, misperceptions, illusions and hallucinations, our recent study provides suggestive evidence that a phenotype characterized by fluctuations of alertness, RBD and VH may be linked to persistent DMN activity. Further work characterizing the role of DAN and VAN, which might be particularly affected in DLB and PDD may provide further clues to the potential contribution of attentional dysfunction and cognitive fluctuations to VH in these conditions.

## 8. Conclusions

Patients with PD or the associated Lewy body dementias of PDD/DLB display a wide range of visual symptoms ranging from visuo-perceptive difficulties, blurred vision, and diplopia through to illusionary experiences and frank VH. These symptoms have significant sequelae for both patients and their carers; the presence of VH is associated with distress and poorer quality of life and an increased likelihood for institutionalization and even death [45].

We have presented a discussion on the potential aetiological mechanisms which give rise to VH in these conditions and these encompass changes in neurotransmitter function, pathological changes in the visual system and the potential importance of attentional dysfunction. We proffer a hypothesis that VH are perhaps, in part, mediated via DMN dysfunction and attentional networks such as the DMN and DAN and VAN and thus would direct that further research in this area is needed. In addition, elicitation of reliable VH in an experimental setting would allow the development of powerful new paradigms to explore the underlying pathophysiology of VH in PD and Lewy body dementias. In the study by Taylor et al. [106] occipital transcranial magnetic stimulation provided an external method to generate VH in DLB although these occurred infrequently and only in a minority of patients. Another technique, developed by our group, using virtual reality presentation (Box 3) may provide a promising new approach to reliably testing VH in an experimental setting.

Table 1
Definitions and glossary of VH and other visual phenomena

| Visual phenomenon | Definition |
|---|---|
| Illusions | Misinterpretation of images. |
| Pareidolias | Complex visual illusions involving ambiguous forms that are perceived as meaningful object as faces-people. |
| Visual hallucinations | Perceptions of images not present in the visual field i.e. images in the absence of external sensory stimulus. Thus VH are considered a "unitary pathological symptom distinct from illusion". |
| Phosphenes | Unstructured lights such as flashes, sparkles, colored dots, zig-zag lines or rainbows, black and white or colored, static or moving. |
| Photopsias | Geometric elementary structured images, often recurring in a repetitive form. |
| Visual distortions | Illusions, as the perceived image consists of the distortion of an image which is present or was present in the visual field of the subject. |
| Visual allesthesias | Condition in which visual images are transposed from one half of the visual field to the other, either vertically or horizontally. |
| Micropsia | Reduced size of the object. |
| Macropsia | Enlarged object. |
| Pelopsia | The object appears closer than actual. |
| Teleopsia or Telopsia | The object appears farther than actual. |
| Metamorphopsia | The distortion of objects or figures, like enlargement of particulars, e.g. elongated necks, fanglike dentures. |
| Kinetopia | The illusion of movement of a static object. |
| Palinopsia | The visual perseveration or recurrent appearance of a visual image after the stimulus has disappeared. |
| Poliopia | The multiplication of the visual image in the visual field. |
| Tassellopsies (or Teicopsies) | Hallucinations consisting of the perception of brick-like textures in the visual field and include fortification spectra and heat waves appearing in migraine. |
| Dendropsies | Hallucinations of tree branches (dendron). |
| Hypnagogic hallucinations | Appearing when falling asleep. |
| Hypnopompic hallucinations | Appearing when waking from sleep. |
| Simple hallucinations | Phenomena like photopsia or perception of static images. |
| Complex hallucinations | Kinetic/kinematic with preserved or disturbed insight. |
| Extracampine hallucinations | Sensation of presence of somebody/something (e.g. guardian angel) at the border or external to the visual field. |
| Serotine misinterpretations | These terms refer to occurrence of illusions (i.e. moving leaves interpreted as people) in the late afternoon / early evening (serotine). This disorder is mostly described in late AD and is an essential part of the "sundowning" phenomenon and is also defined as Pareidolia. |
| Movement hallucinations | Sensation of passage (brief vision of persons or animals passing on the sides of the visual field). |
| Minor forms of VH | Include: presence of extracampine or presence hallucinations; passage hallucinations; or as sometimes described (although phenomenological incorrect) illusions. |
| Formed hallucinations | Formed hallucinations with various contents (persons, animals, objects, interacting with each other and with the patient in complex scenes). |
| Blurred or formed | Blurred hallucinations are described as indefinite or not fully formed images, like presence and passage VH. Formed images can be inanimate or animate figures, and can be static or moving in complex interactions. |
| Moving images/Kinetic and kinematic (movie like) | Can be simple or minor, blurred, complex or formed. |
| Simple or complex | The classification in simple/complex VH can be confounding because the terms are sometimes used to describe VH characterized by preserved vs. disturbed insight. |
| Benign or malignant | VH can be described as benign or malignant. The terms have been applied in the characterization of VH in 1) the context of retained insight or disrupted insight 2) the proneness of the disturbance to remain stable or to progress with PD 3) labelling the nature of the disturbance as mild (not bothersome for the patient) or severe (affecting patient quality of life). However, this classification criterion has been challenged because VH severity and frequency tend to progress, and thus "the term benign hallucinations of PD should be considered generally unsound and dropped from operative vocabulary" [45]. |
| Early or late | VH in PD have also been tentatively classified as early, appearing within 5 years from the onset of PD or late. This classification too has been challenged. |
